# Indexical and linguistic processing by 12-month-olds: Discrimination of speaker, accent and vowel differences

**DOI:** 10.1371/journal.pone.0176762

**Published:** 2017-05-17

**Authors:** Karen E. Mulak, Cory D. Bonn, Kateřina Chládková, Richard N. Aslin, Paola Escudero

**Affiliations:** 1 The MARCS Institute for Brain, Behaviour and Development, Western Sydney University, Penrith, New South Wales, Australia; 2 Australian Research Council Centre of Excellence for the Dynamics of Language, Western Sydney University, Penrith, New South Wales, Australia; 3 Department of Brain & Cognitive Sciences, University of Rochester, Rochester, New York, United States of America; 4 Amsterdam Center for Language and Communication, University of Amsterdam, Amsterdam, Netherlands; Utrecht University, NETHERLANDS

## Abstract

Infants preferentially discriminate between speech tokens that cross native category boundaries prior to acquiring a large receptive vocabulary, implying a major role for unsupervised distributional learning strategies in phoneme acquisition in the first year of life. Multiple sources of between-speaker variability contribute to children’s language input and thus complicate the problem of distributional learning. Adults resolve this type of indexical variability by adjusting their speech processing for individual speakers. For infants to handle indexical variation in the same way, they must be sensitive to both linguistic and indexical cues. To assess infants’ sensitivity to and relative weighting of indexical and linguistic cues, we familiarized 12-month-old infants to tokens of a vowel produced by one speaker, and tested their listening preference to trials containing a vowel category change produced by the same speaker (linguistic information), and the same vowel category produced by another speaker of the same or a different accent (indexical information). Infants noticed linguistic and indexical differences, suggesting that both are salient in infant speech processing. Future research should explore how infants weight these cues in a distributional learning context that contains both phonetic and indexical variation.

## Introduction

The primary mechanism by which humans come to learn and discriminate tokens of speech sounds (i.e., phonetic tokens) sampled across native speech-sound category boundaries has been proposed to be unsupervised distributional learning over the raw acoustic input [1–5]. Unfortunately for infants, unsupervised distributional learning of the acoustic environment is a difficult computational problem, as variability in the environment is conditionally dependent upon vocal tract differences among talkers who produce the input presented to infant listeners. Failure to consider these differences leads to unresolvable overlap that does not allow for reliable extrication of the distributions of many categories [6]. However, infants may learn to systematically accommodate these sources of talker variability *if* they can discriminate among them and subsequently learn how to adjust their speech-recognition mechanisms accordingly. We focus here on infants’ ability to discriminate among and weight indexical and linguistic sources of variability in the speech signal.

**Fig 1:**
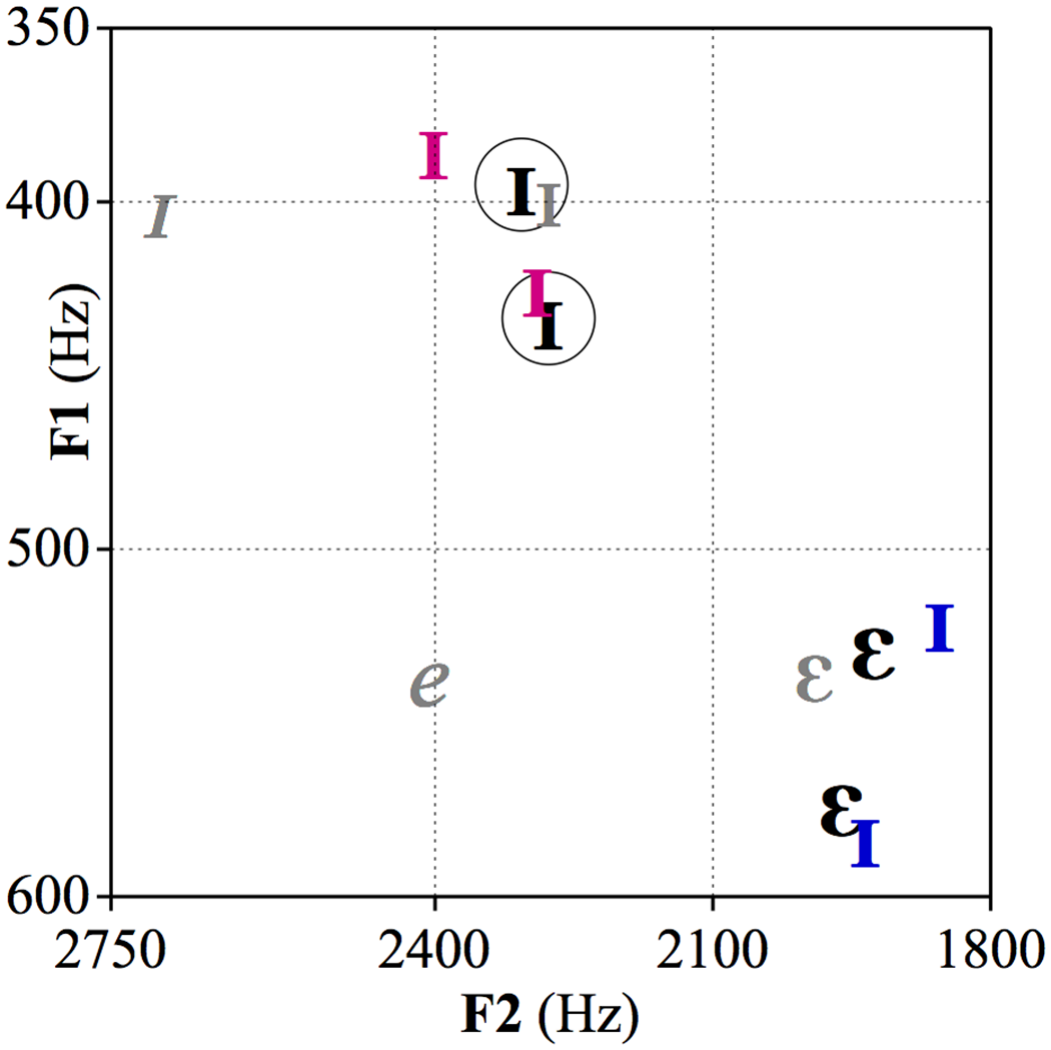
First (F1) and second (F2) formant values of the familiarization and test stimuli in the present study. Black = First North Holland Dutch (NHD) speaker. Circled /ɪ/ tokens were used in familiarization and Same test trial. Uncircled /ɛ/ tokens were used in Vowel change test trial. Magenta /ɪ/ = Second NHD speaker. Tokens are those used in Speaker change trial. Blue /ɪ/ = East Flemish Dutch speaker. Tokens are those used in Accent change trial. Grey values represent mean values of AusE (italicized) and NHD (unitalicized) measured in the Australian English and NHD female population by Cox [11] and Adank et al.[12], respectively.

The variability in the acoustic environment mostly stems from anatomical vocal tract differences between speakers who produce the speech sounds in question. During vowel production, the air passing though the vibrating vocal folds produces a carrier signal that gets further modified in the upper parts of the vowel tract. The positions of articulators, such as the tongue or lips, results in specific frequencies at which the carrier signal resonates. Different steady-state vowel qualities are most reliably cued by their first (F1), second (F2) and third (F3) resonating (or, formant) frequencies which roughly reflect the shape and size of the articulatory space vis-à-vis the vertical position (height) of the tongue within the mouth (F1), the horizontal position (backness) of the tongue (F2), and lip rounding (F3). For instance, vowel F1 typically ranges between 200 and 1200 Hz and is inversely related to tongue height: a vowel like /i/ is produced with a high tongue position and it has a low F1. However, as mentioned above, formant values for a particular vowel are largely influenced by the anatomical properties of the speaker’s vocal tract, resulting in an overlap of different vowel qualities in the infants’ auditory environment when they are produced by different speakers (see e.g., [7]). These values are further affected by idiolectal differences, whereby speakers within the same speech community differ in their mean and range of frequency values for a given vowel (e.g., [8–10]). Across speech communities, such as languages, accents and sociolects, systematic variation can occur, to the point that vowel category formants in a non-native accent can completely overlap with formants for a different vowel in the listeners’ native accent (e.g., see Fig 1).

Together, these sources of variability mean that raw formant frequencies do not reliably cue vowel category membership across speakers when there are large differences in vocal tract size. Raw formant frequencies also cannot reliably cue vowel qualities when faced with between-group variations in speech, such as those between speakers of two different accents of a language, and instead add another dimension of variability that the listener must resolve for successful perception. Yet, infants seem to recognize equivalence classes of vowels in spite of indexical (and other phonemically irrelevant) variation [13,14]. Thus, the question of how infants become able to recognize speech categories despite the presence of ubiquitous between-speaker acoustic-phonetic variability does not have a straightforward answer.

In the past, researchers have proposed that listeners resolve this between-speaker variation by normalizing the incoming speech signal in order to parse out the invariant cues that allow for reliable identification of the linguistic content. Many attempts have been undertaken to identify these invariant cues, and researchers have proposed that they emerge through ratios between formants (see [15] for a review). While this approach has a strong intuitive appeal, no proposal has been able to fully account for perceptual findings—for instance, models that incorporate F0 as part of invariant structure (e.g., [16–18, 15]) cannot account for listeners’ identification of whispered vowels, for which F0 is absent. Due to their limited success, these models have fallen out of favor in recent years.

More recently, it has been proposed that listeners instead handle variability by partitioning between- and within-speaker variability via the adjustment of prior conditional probabilities related to speaker qualities (e.g., [19]). By this process, certain properties of the speaker are taken into account, thereby eliminating between-speaker variance, and the distribution of expected acoustic realizations is adjusted accordingly. For example, if a speaker is known to be female, the centroid of the expected acoustic realization in the vowel space will be shifted away from distributions associated with male speakers. The same principle applies to other systematic sources of variability in production, such as regional accent. This means that conditioning on indexical variables effectively reduces within-category variance, thereby reducing the complexity of the perceptual problem.

Fundamental to this approach, however, is an underlying knowledge of variability in production that can be attributed to different properties of the speech signal. In order to contextualize the speech signal, one needs to be intimately familiar with the range and kinds of variability that can be attributable to speaker properties and regional accent (i.e. indexical properties) versus linguistic properties. Thus, it is immediately apparent that successful speech recognition requires a sophisticated set of skills for making complex inferences. Indeed, Kleinschmidt and Jaeger [19] propose that this process involves a degree of constant learning and adaption to account for new speakers, idiolects, accents, dialects, and sociolinguistic change. The question naturally arises of how children learning their first language are able to handle this task. In order to appropriately partition sources of variance in the speech signal, they must be familiar with the kinds and amount of variance attributed to speaker, accent, and linguistic properties. Where they do not have this knowledge, the variability present in a distribution of tokens could be incorrectly attributed one of these three properties or otherwise interfere with the process of speech recognition.

Research on the development of speech processing shows a progression in infants’ ability to attribute variability to different properties of speakers. Newborns show remarkable sensitivity to linguistic information in speech sound properties [20]. By two months, infants demonstrate some ability to recognize speech across speakers: When familiarized to a syllable produced by three speakers, infants can notice a syllable change produced by the same three speakers [21]. However, infants initially struggle when it comes to recognizing the same words produced by two very different speakers. Houston and Jusczyk [22] found that while 7.5-month-olds could recognize words produced by two speakers of the same gender, only 10.5-month-olds could recognize words produced between genders. Subsequent research found that 7.5-month-olds could recognize words between genders if they first received exposure to passages of fluent natural speech, and were then tested on their recognition of isolated words embedded in the passages [23]. The ability to recognize words between different accents occurs even later, with word recognition abilities emerging by 12 months (e.g., [24]), and word identification emerging in the second year [25,26]. Infants’ earlier failures imply that the indexical variation may not have been recognized as such, and instead may have been treated as linguistic variation. This in turn would result in a failure to partition the speech signal by speaker properties, and consequently lead to the variance from indexical components having an additive effect to the already existing linguistic variation.

In sum, although very young infants can utilize non-linguistic information available in the speech signal to differentiate between speakers (e.g., in their ability to recognize their mother’s voice: [27], or differentiate between voices of different strangers: [21,28]), they appear unable to use this variation appropriately in certain circumstances or tasks (e.g. they may wrongly attribute it to linguistic variation as in the word-identification tasks summarized above). The question therefore remains whether infants attend to structured indexical information in the input for purposes of learning new speech sound categories from their frequency distributions. Attention to indexical information could potentially help infants extract phonemically relevant information during speech sound learning, by signaling that certain properties of speech are partitioned into non-linguistic categories that serve as “contexts” for extracting the linguistically relevant sources of variation.

A first step in identifying possible strategies infants could use to identify relevant sources of variation is to explore the relative salience of indexical (speaker, accent) and linguistic (phonemic distinction) cues available to infants in passive exposure. To our knowledge, no prior experiment has directly compared infant attention to these cues while holding the task for the infant constant. If infants can track the identity of speakers as well as track the linguistic quality of speech tokens, it would indicate the possibility that infants adjust to speaker differences in the acoustic realizations of vowels, which allows them to reliably infer the location of vowel distributions in the multidimensional acoustic space.

In the present study, we tested in detail infants’ sensitivity to both indexical and linguistic differences in speech sounds. The availability of both indexical and linguistic cues may help organize statistical information about vowels and speech categories more generally (e.g., in adulthood: [29]). In spite of tremendous variability in phoneme realizations across speakers of a specific accent, and even greater variability across speakers with different accents, adults seem able to rapidly accommodate both speaker and accent variability with ease when lexical information is available [29–31]. However, adult listeners accommodate speaker but not accent variability when higher order information (from the lexicon, context or through feedback) is unavailable [32,33].

To examine infants’ sensitivity to linguistic and indexical cues at a point when they leverage their experience to handle novel situations, (and not merely learn unmodifiable, static representations), we tested infants past the point at which they become attuned to the native vowel categories of their language (6–8 months: e.g., [13]), past the point at which they demonstrate an ability to recognize speech across speakers (10.5 months: [22]), and when they are at the point of demonstrating an ability to recognize speech across accents (12 months: [24]). We familiarized 12-month-olds to tokens of a vowel (/ɪ/) produced by a native female speaker of North Holland Dutch (NHD), and compared—in a mixed between- and within-subject design—infants’ looking time to four types of test trials: (1) *Speaker change*: introducing an indexical change (via a change to another native female speaker of NHD, (2) *Accent change*: a change in speaker to a female speaker of East Flemish Dutch: EFD, (3) *Vowel change*: a linguistic change (via a change in vowel [to /ɛ/] with speaker held constant, and (4) *No change*: test trials containing the same tokens as in familiarization. Attention to these indexical and linguistic differences would indicate the possibility that these cues are available to infants for sociolinguistic as well as linguistic purposes. We tested NHD-learning and Australian English (AusE)-learning infants in order to investigate whether the ability to process both indexical and linguistic cues applies cross-linguistically, regardless of variation in linguistic properties across languages that contain different phonemes or different realizations of the same phonemes.

## Materials and method

### Participants

Participants were thirty-seven 12-month-old infants from households in Amsterdam, the Netherlands, where NHD was spoken (Speaker-change condition: 16 participants, 8 females, *M*_age_: 12.20 months, *SD*_age_ = 0.43 months; Accent-change condition: 21 participants, 14 females, *M*_age_ = 12.27 months, *SD*_age_ = 0.35 months) and thirty-seven 12-month-old infants from households in Sydney, Australia, where AusE was spoken (Speaker-change condition: 16 participants, 8 females, *M*_age_ = 11.85 months, *SD*_age_ = 0.58 months; Accent-change condition: 20 participants, 10 females, *M*_age_ = 11.96 months, *SD*_age_ = 0.57 months). A participant group size of 36 per language group was decided upon based on the sample size used in previous work implementing a similar task that investigated infants’ ability to recognize speech across indexical cues [22]. Due to experimenter error, more participants in the AusE sample received a Speaker change as their Indexical change trial, than an Accent change (see the Procedure section, below). This was then matched in the NHD sample. However, the data were subsequently analyzed using a mixed effects model, which is not influenced by differences in sample sizes across groups [34]. NHD-learning infants were tested at the University of Amsterdam and AusE-learning infants were tested at Western Sydney University. Data from 33 additional infants were collected but not included in analysis due to fussiness or disinterest (*N*_AusE_ = 18, *N*_Dutch_ = 10), technical issues (*N*_AusE_ = 3, *N*_Dutch_ = 1), or experimentor error (*N*_AusE_ = 2).

### Stimuli

Infants were presented with naturally produced Dutch vowels extracted from read sentences. The vowels were selected from a larger corpus of Dutch vowels (as reported in [12]). We chose the vowels /ɪ/ and /ɛ/ (as in “pit” and “pet”) because they have large variation in their acoustic properties across both Dutch and English accents, thus providing a realistic context in which speaker and accent variability would be behaviorally relevant. While both AusE and NHD have the vowel /ɪ/, the vowel is fronted in AusE relative to NHD [11,12]. However, categorization results demonstrate that at least in adults, NHD /ɪ/ is typically categorized as /ɪ/ by AusE listeners [35]. Acoustic analysis [11] suggests that in place of /ɛ/ Australian English has /e/, and that this vowel is more acoustically similar to NHD /ɪ/ than to NHD /ɛ/. Nevertheless, 15-month-old AusE infants have been found to discriminate vowel contrasts on the basis of magnitude of acoustic difference rather than adherence to native vowel categories [36]. Thus, we predicted that the acoustically distinct /ɪ/–/ɛ/ contrast should be discriminable for AusE-learning infants as well as NHD-learning infants, despite it being a non-native contrast for the former.

The Dutch vowels presented to the infants were produced by two female speakers of the same Dutch accent (NHD) and by a female speaker of a different Dutch accent (EFD). Fig 1 shows the F1 and F2 values of these vowels, and Tables 1 and 2 respectively show the raw acoustic values, and differences in mean values of the stimuli used. As can be observed, acoustic analysis of the stimuli confirmed that F1 and F2 values of /ɪ/ are more similar across speakers of the same accent than across speakers of different accents. Importantly, the values of EFD /ɪ/ were closer to those of NHD /ɛ/ than to NHD /ɪ/. The tables further show that the measures of voice quality, i.e. F0 and energy in the high frequency range between 5000 and 8000 Hz, in the Speaker- and Accent-change stimuli differ from the Familiarization stimuli numerically more than those in the Vowel-change stimuli.

**Table 1:** Raw and averaged acoustic values of the stimuli used. The “Stimulus” heading shows the speaker (NHD1, NHD2 or EFD1), vowel (/ɪ/ or /ɛ/), and token.

	Stimulus			Duration (ms)	F0 (Hz)	Energy in 5000–8000 Hz (dB)	F1 (Hz)	F2 (Hz)	F3 (Hz)
	Speaker	Vowel	Token						
Familiarized/No change	NHD1	/ɪ/	1	60	208	-4.1	342	2344	2898
	NHD1	/ɪ/	2	56	216	-5.1	387	2337	3023
	Mean			58	212	-4.6	364.5	2340.5	2960.5
Vowel change	NHD1	/ɛ/	1	57	209	-0.2	487	1975	2872
	NHD1	/ɛ/	2	57	194	0.1	432	1976	2800
	Mean			57	201.5	-0.05	459.5	1975.5	2836
Speaker change	NHD2	/ɪ/	1	60	205	-11.1	381	2423	3115
	NHD2	/ɪ/	2	60	252	-10.9	341	2496	2973
	Mean			60	228.5	-11	361	2459.5	3044
Accent change	EFD1	/ɪ/	1	55	256	-0.1	506	1797	2893
	EFD1	/ɪ/	2	57	303	5.4	581	1947	3050
	Mean			56	279.5	2.65	543.5	1872	2971.5

**Table 2:** Difference in acoustic values between the two Familiarization/No change tokens, and the differences between the average acoustic values of the two test tokens for each change type minus the average acoustic values of the two Familiarization/No change tokens.

	Duration (ms)	F0 (Hz)	Energy in 5000–8000 Hz (dB)	F1 (Hz)	F2 (Hz)	F3 (Hz)
	Difference between the two Familiarization/No change tokens					
Familiarization/No change	-4	8.0	-1.00	45.0	-7.0	125.0
	Differences between the average values for the two Change trial tokens and the two Familiarization/No change trial tokens					
Vowel change—Familiarization/No change	-1	-10.5	4.55	95.0	-365.0	-124.5
Speaker change—Familiarization/No change	2	16.5	-6.40	-3.5	119.0	83.5
Accent change—Familiarization/No change	-2	67.5	7.25	179.0	-468.5	11.0

### Apparatus and setup

Participants’ gaze was measured using a Tobii X120 eyetracker at Western Sydney University, and a Tobii T120 eyetracker at the University of Amsterdam (Tobii Technology, Danderyd, Sweden), both sampling at 120 Hz. These eyetrackers are accurate within 0.5° and have a 0.2° compensation error for head movements. They implement both dark-pupil and bright-pupil technology to minimize data loss, and track both eyes simultaneously, which allows for data collection even when one eye is not being tracked. This binocular tracking also allows for correction of drift through continuous averaging of drift effects between the two eyes.

The testing rooms in both locations were set up similarly, according to the hardware and software available at each location. The testing room in Sydney was set up with a 19-in. ViewSonic monitor (Brea, California, United States) 50 cm behind the front of the eyetracker, and with its lower edge positioned 21.5 cm above the table on which the eyetracker sat. A single area of interest (AOI) was drawn to cover the entire monitor, as we were interested in whether children were or were not looking at the monitor. The AOI measured 37.7 cm x 30.3 cm, which subtended a viewing angle of 18.1° x 14.6° when the participant was seated 70 cm away from the front of the eyetracker. Two adjacent Edirol MA-15D speakers (Roland Corporation, Hamatsu, Japan) were centered to the right of the monitor such that the left edge of the left speaker was 7 cm to the right of the right edge of the monitor. A Logitech web camera (Lausanne, Switzerland) was placed on top of the monitor, allowing the experimenter to view the participant from the adjoining room and verify that the participant’s gaze was being tracked when it was oriented toward the screen. The camera did not transmit an audio signal, so experimenters were blind to the experimental condition.

The testing room at the University of Amsterdam implemented the 17-in monitor built into the Tobii T120 eye-tracker, which is permanently fixed directly above the eyetracker. A single AOI was drawn to cover the entire monitor, which measured 33.8 cm x 27.0 cm. This subtended a viewing angle of 29.1° x 23.5° when the participant was seated 65 cm away from the front of the eyetracker, which was the recommended distance for the eyetracker. Although the viewing angles differed between setups, it was the auditory, and not the visual stimulus that was the critical stimulus, and the paradigm measured attention to the critical stimulus via gaze to and away from the entire monitor, rather than fine-grained looking within the monitor perimeter. Two adjacent Tangent EVO-E4 speakers (Tangent A/S, Herning, Denmark) were centered to the right of the monitor such that the left edge of the left speaker was 7 cm to the right of the right edge of the monitor. A web camera built into the Tobii T120 eyetracker and centered above the monitor allowed the experimenter to view the participant from the adjoining room and verify that the participant’s gaze was being tracked when it was oriented toward the screen. The camera was muted to ensure experimenters were blind to the experimental condition.

### Procedure

This study was undertaken with approval from both the Western Sydney University Human Research Ethics Committee (approval H9373) and the University of Amsterdam Commissie Ethiek (approval 2014–4). Prior to participation, caregivers provided informed written consent in accordance with human research ethical standards at Western Sydney University and the University of Amsterdam. Participants were seated on their caregiver’s lap so that their eyes were approximately 65–70 cm from the front of the eyetracker. For the duration of the study, caregivers listened to a mixture of music and speech through circumaural headphones so that caregivers were unable to hear the experimental stimuli. Caregivers were asked to look down or to the side for the duration of the experiment to ensure that their eyes were not tracked instead of the child’s.

Before testing began, each participant’s gaze was calibrated to a dynamic cartoon paired with sound, presented nine times so that gaze position spanned a 3x3 grid on the monitor. The cartoons measured 5 cm x 5 cm and were presented with Tobii Studio. The experimenter determined participants to be looking at the calibration stimuli when their gaze was fixed at a point on the screen corresponding to or in close proximity to the calibration object.

Following calibration, participants completed a serial preference procedure in which we measured infants’ looking times to trials composed of strings of vowel tokens. Stimuli were presented using E-Prime (version 2.0, Psychology Software Tools, Inc., Sharpsburg, Pennsylvania, United States). Infants first heard eight familiarization trials containing eight repetitions of each of two tokens of the vowel /ɪ/ (as in KIT), produced by one of the female NHD speakers (depicted as the two circled /ɪ/-tokens in Fig 1). Each familiarization trial presented the 16 tokens in a fixed random order with a 750 ms inter-stimulus-interval, resulting in a trial duration of 13 sec. Each of the eight familiarization trials had a unique randomization of the 16 tokens, and the order in which participants were exposed to the eight familiarization trials was randomized. The familiarization phase lasted approximately two minutes.

**Fig 2:**
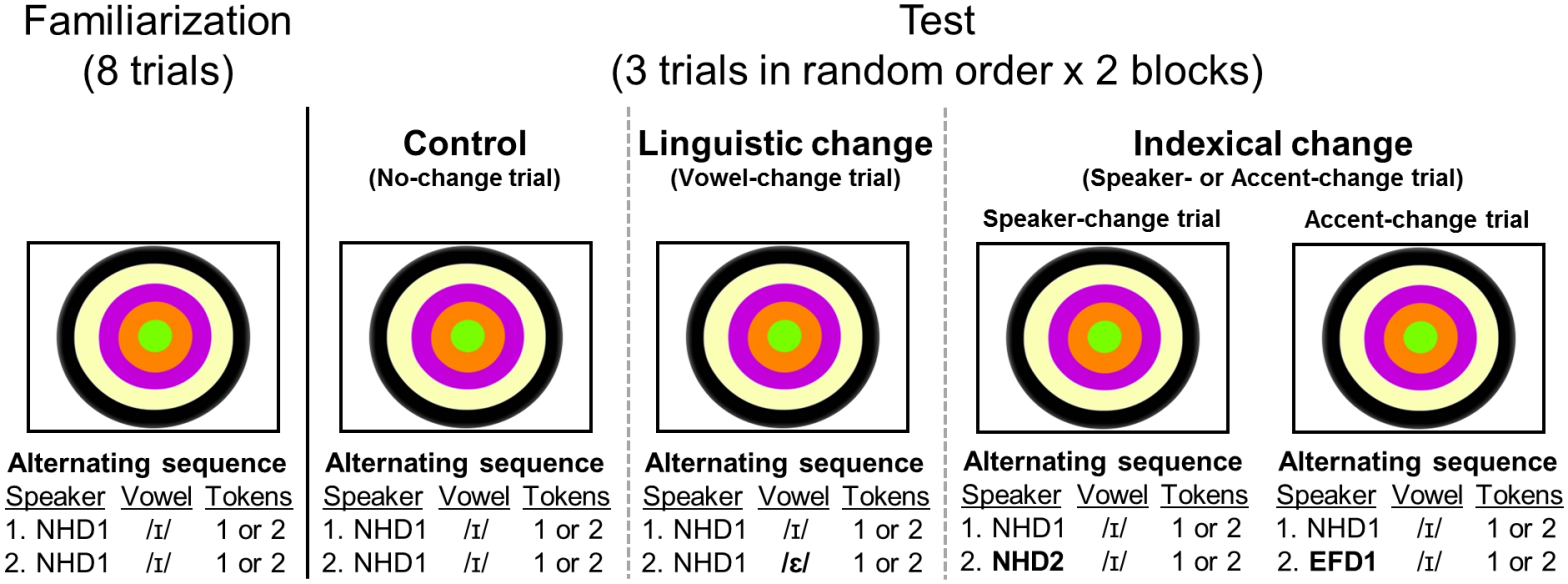
A schematic of the design. Each participant was exposed to eight familiarization trials presented in random order, and three test trials presented in random order. Tokens were produced by one of two female speakers of North Holland Dutch (NHD1, NHD2) or a female speaker of East Flemish Dutch (EFD). Participants heard tokens in an alternating sequence as indicated, with selection of token 1 or token 2 in each instance randomized once.

After familiarization, infants were presented with three test trials in random order: A No-change trial, a Vowel-change trial, and an Indexical-change trial, which involved either a speaker change (Speaker-change trial), or a speaker and accent change (Accent-change trial). Examples of these trials can be seen in Fig 2. In each test trial, two tokens of the test stimulus were alternated with the two tokens of the familiarization stimulus eight times. While the alternating order of the familiarization stimulus and test stimulus was fixed, which of the two tokens played for each stimulus was randomized once for each test trial, with each token occurring four times. Thus, as in the familiarization, participants heard 16 total vowel tokens, there was a 750 ms ISI between tokens, and each test trial lasted approximately 13 seconds. The No-change trial served as the control. In this trial, the alternating stimuli were the same two tokens of /ɪ/ as those used in the familiarization. In the Vowel-change trial, familiarization stimuli were alternated with two tokens of the vowel /ɛ/ (as in DRESS) produced by the familiarization speaker. The Indexical-change trial introduced either a speaker change (Speaker-change trial) or a speaker and accent change (Accent-change trial), that is, Indexical change refers to a trial that contained a change in speaker identity. For the Speaker-change trial, the familiarization stimuli were alternated with two tokens of the familiarization vowel /ɪ/, but produced by a different female speaker of the same NHD accent, and were alternated with two tokens of the vowel /ɪ/ produced by a different speaker of a different accent of Dutch (EFD).

## Results

To determine whether infants detected linguistic vowel changes and indexical speaker and accent changes and the relative salience of these changes, we fit a Bayesian, multilevel linear model to participants’ proportion fixation time to each test trial.

### Familiarization results

**Fig 3:**
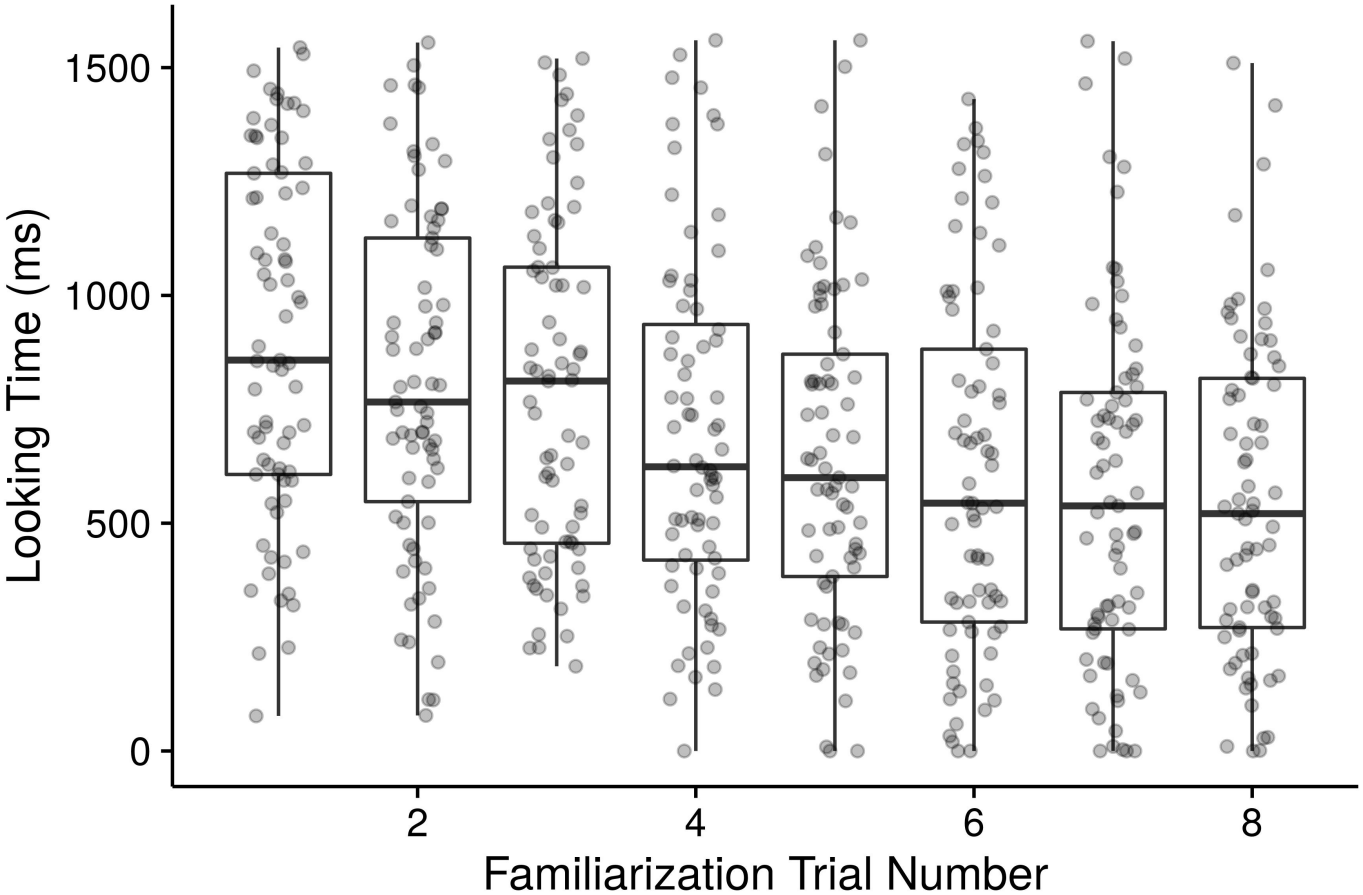
Looking times during familiarization. Individual points represent raw looking times for each infant and boxplots represent the median, interquartile (boxes) and 1.5*interquartile range (whiskers).

First, we visually and quantitatively explored infants’ looking behavior in the eight familiarization trials (see Fig 3) and extracted the fitted parameters for each infant from a linear regression model in the following steps.

Data were rescaled from milliseconds to be between 0 and 1, inclusive. The data showed considerable non-normality, given both the expected negative skew of infant looking times in general and more unusual boundedness on trial length specific to this design. To systematically choose the proper transform, we compared the AIC values from full maximum-likelihood models fitted in lme4 in R using 5 potential transforms: (1) the identity (no transform), (2) the log transform (as recommended in [37] for head-turn preference procedure), (3) the square-root transform, (4) the empirical logit transform (that is, *log* (p+0.5)/(1.5 –p)), and (5) the arcsine-square-root transform. In the models, we included Trial Number and the Intercept as fixed effects, with random intercepts and slopes by infant. The arcsine-square-root transform received the best (lowest) AIC score, so we selected that as the appropriate transform for the rest of the analysis, including test trials. Visual inspection of the residuals of each model confirmed that it removed the most non-normalities.

We then refitted the best-fit model using the rstanarm package [38], a tool for easily fitting Bayesian models using stan [39]. We chose Bayesian methods for inference because it allows for easier handling of missing data (common with infants, particularly in later trials). We then extracted the median of the posterior distributions of the random slopes and intercepts for each infant to use as covariates in the model of the test data.

### Results: Test

**Fig 4:**
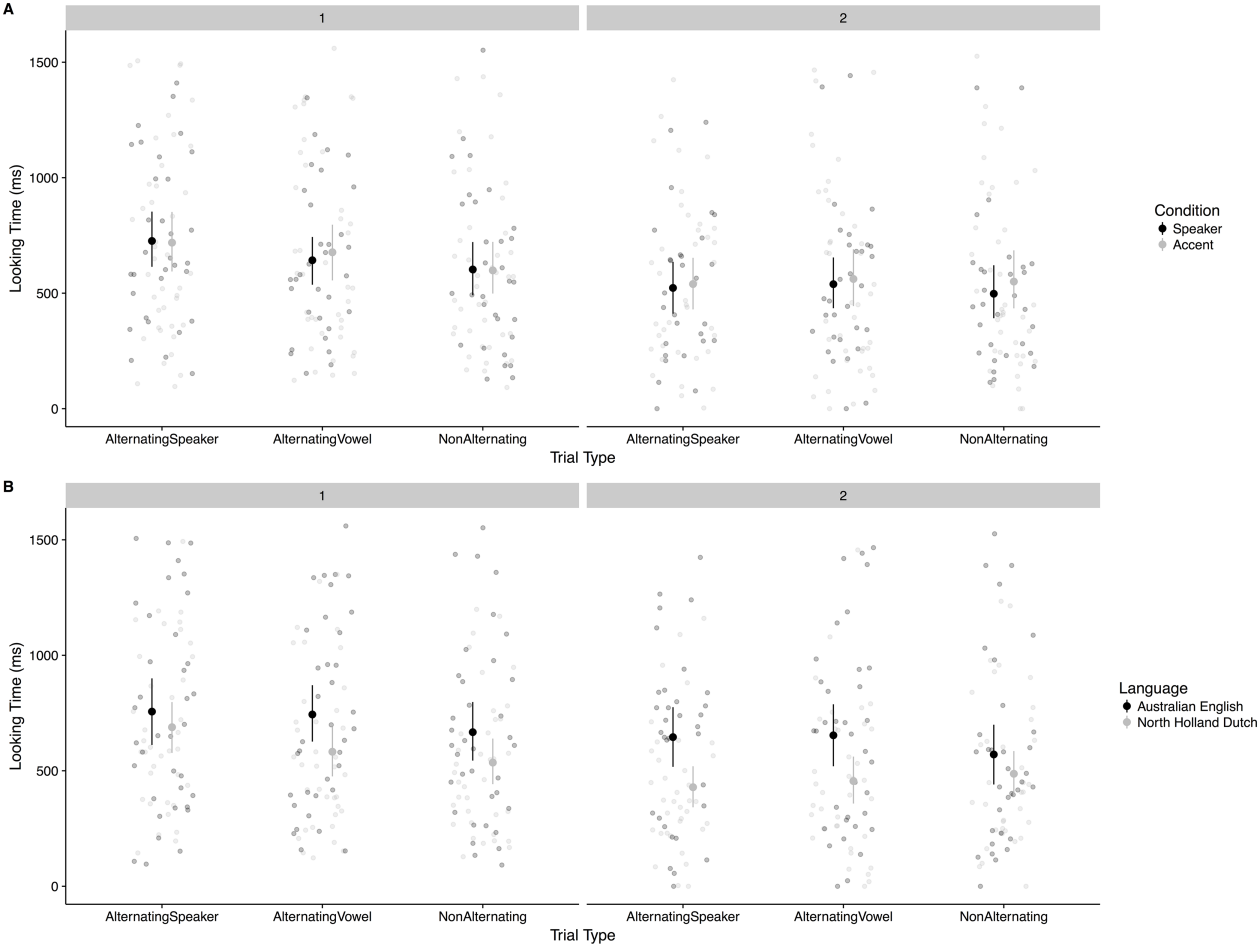
Test trial looking times. Each panel represents a Trial Block. Error bars are 95% CI calculated via bootstrap in ggplot2.

To explore the test data before running the full model, we examined scatterplots of the individual data points to make sure the data appeared similarly distributed to the familiarization trials as well as to examine whether there could be differences in Condition assignment between the two indexical change types (Speaker-change vs. Accent-change; see Fig 4a) and Language (see Fig 4b). The infants’ behavior in the two stimulus conditions appeared nearly identical, so we combined these groups of infants together into a single Indexical change group. In addition, in preliminary models estimated with lme4 without examining effect significance, we found that the variance predicted by Language Group was largely soaked up by the parameters estimated in the Familiarization model (that is, there was a difference in average looking time of unknown origin across groups), so we also combined Language groups for modeling simplicity.

For modeling the test phase data, we entered the following coefficients into a Bayesian, multilevel model estimated using rstanarm: (1) an intercept, (2) Trial Block, (3) Trial Type, (4) the Trial Block by Trial Type interaction, (5) Familiarization Slope, (6) Familiarization Intercept, and a random slope and intercept by Trial Block for each infant. All predictors were centered. Trial Type was simple coded (centered dummy codes) with No-change trials assigned as the reference Type. Our decisions about the presence of effects was based on whether the 95% Highest Density Interval (HDI) for the estimated coefficients included zero.

**Fig 5:**
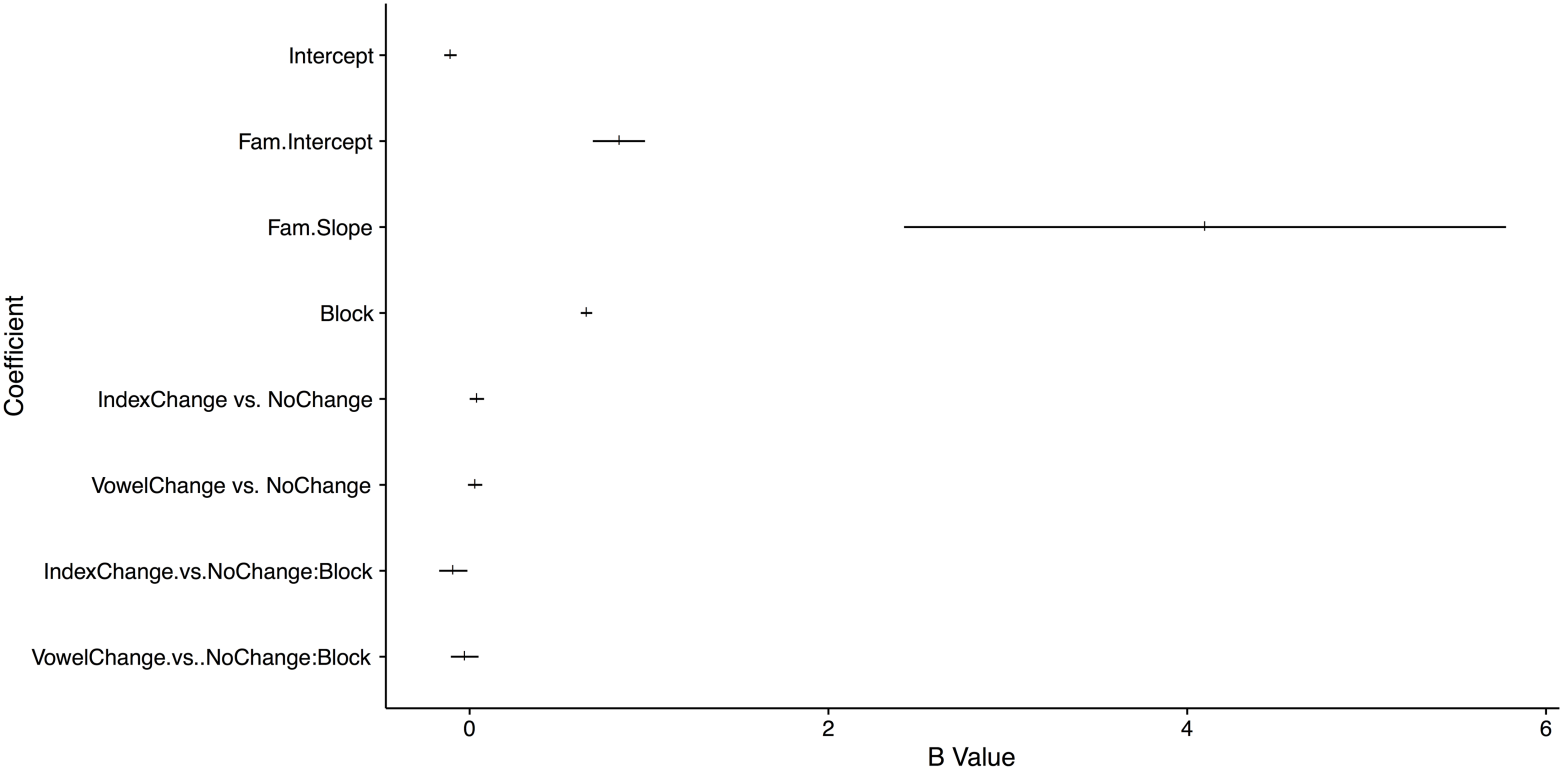
Regression table in graphical form (c.f. Table 3). The 95% HDI and the posterior medians are indicated for each coefficient (50% Interval omitted due to visibility constraints).

The posterior distributions over the parameters are summarized in Table 3, with Fig 5 illustrating their relative effect sizes more clearly. Infants’ looking behavior during familiarization predicted looking during test in both their intercept and slope, such that the higher the random intercept at familiarization, the longer infants looked in test; this coefficient thus accounts for overall interest of the infant. The higher the random slope during familiarization, the higher the looking time at test; and the shallower the decline in attention in familiarization, the more likely infants were to look longer during test. The coefficient for Trial Block indicated an overall decrease in attention across test blocks.

**Table 3:** Summary of the posterior distributions of the coefficients for the test-data model.

	Quantile				
Coefficient	2.5%	25%	50%	75%	97.5%
Intercept	-0.1416	-0.1188	-0.1071	-0.0953	-0.0722
Block	0.6195	0.6406	0.6513	0.6623	0.6831
Familiarization Intercept	0.6867	0.7837	0.8340	0.8825	0.9776
Familiarization Slope	2.4215	3.5207	4.0990	4.6680	5.7771
Indexical change vs. No change	0.0003	0.0267	0.0404	0.0539	0.0805
Vowel change vs. No change	-0.0093	0.0162	0.0299	0.0437	0.0704
Index change vs. No change:Block	-0.1697	-0.1192	-0.0921	-0.0649	-0.0123
Vowel change vs. No change:Block	-0.1045	-0.0536	-0.0270	0.0002	0.0496

The coefficients corresponding to the effect of Trial Type indicated that infants looked longer to the Indexical-change (significant; 95% HDI = [0.0003, 0.805]) and the Vowel-change stimuli (marginal; 95% HDI = [-0.0093, 0.0704]) than to the No-change stimuli. An additional comparison calculated from the posterior distribution failed to show a credible difference between the Indexical change and Vowel-change trial types overall, with both the 95% and 90% intervals containing 0; 95% HDI = [-.008, 0.062].

**Fig 6:**
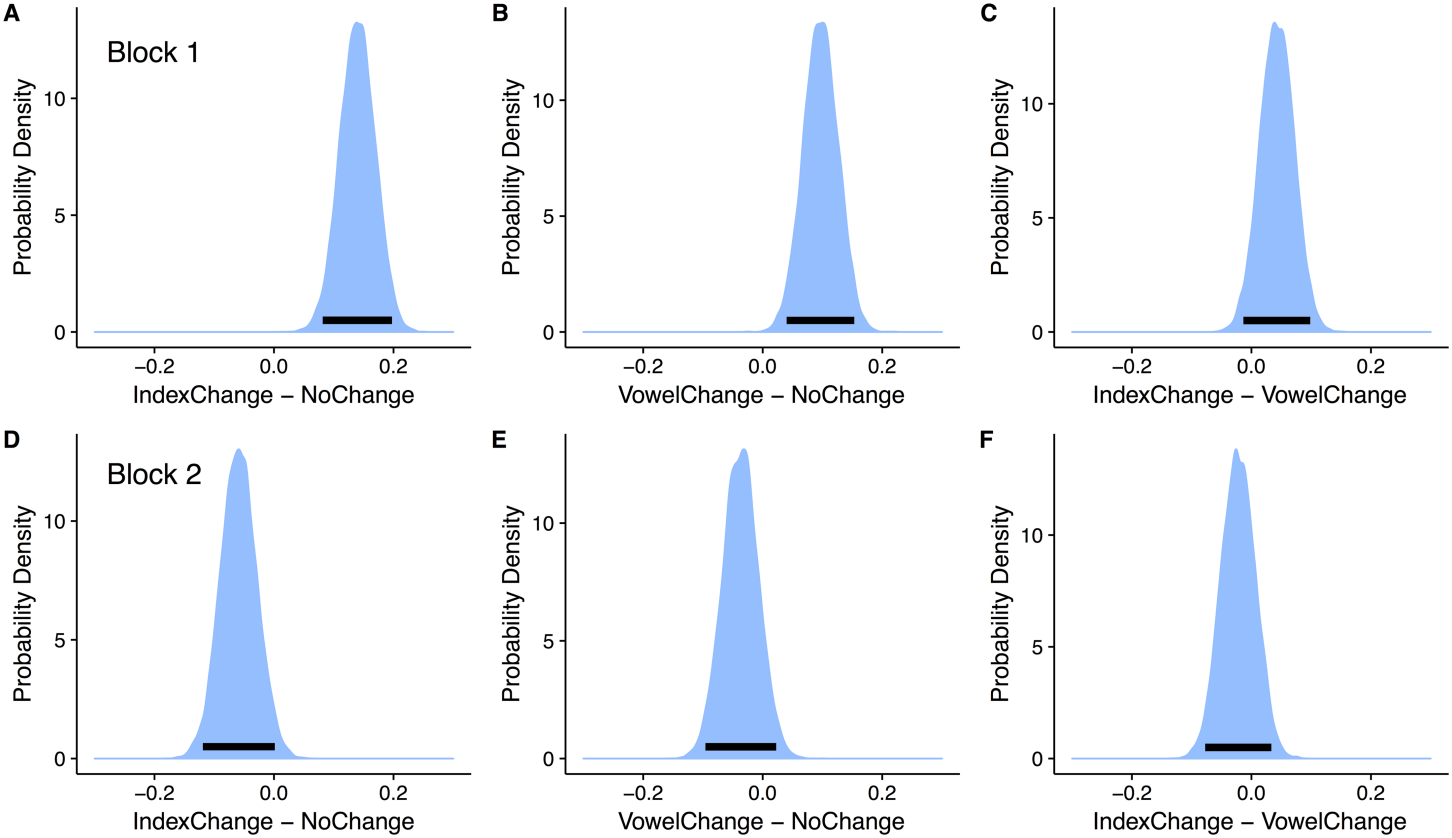
Graphical summary of simple effects. Each panel represents the posterior distribution over looking-time differences for each trial type, for each block. The black bar indicates the 95% HDI.

In addition to an overall decrease in looking time across Test Block, there was an interaction with Trial Type and Test Block, indicating a flattening of the between-condition differences in Block 2, but only for the difference between Indexical-change and No-change types. To examine the complete block-specific differences, we show the full posterior distributions for the simple differences between conditions in Table 4 and Fig 6, adding the additional Indexical-change vs. Vowel-change comparison computed from the posterior samples.

**Table 4:** Table of simple effects. The summary quantiles are indicated for each simple group difference for each trial block.

	Quantile					
Effect	Block	2.5%	25%	50%	75%	97.5%
Indexical change vs. No change	1	0.0817	0.1199	0.1399	0.1596	0.1972
Vowel change vs. No change	1	0.0403	0.0773	0.0969	0.1167	0.1532
Indexical change vs. Vowel change	1	-0.0134	0.0235	0.0428	0.0622	0.0983
Indexical change vs. No change	2	-0.1189	-0.0794	-0.0592	-0.0389	0.0013
Vowel change vs. No change	2	-0.0956	-0.0571	-0.0370	-0.0173	0.0223
Indexical change vs. Vowel change	2	-0.0774	-0.0416	-0.0222	-0.0026	-0.0591

The simple effects show the expected differences between conditions in Block 1, with the looking-time difference between Indexical-change and No-change being qualitatively larger than the difference between Vowel-change and No-change; both simple coefficients’ HDIs exclude zero. The difference between Indexical-change and Vowel-change is not credible (the 95% and 90% HDIs include 0). In Block 2, the difference between Indexical-change and No-change disappears and perhaps reverses, given that the HDI largely is negative (the 90% HDI is below zero, but the 95% HDI contains 0). The difference between Vowel-change and No-change also disappears in Block 2 but does not reverse. Finally, the difference between Indexical-change and Vowel-change also seems to reverse (due to the overall drop in looking time to Indexical-change trials), but not credibly (the 95% and 90% HDIs include 0).

## Discussion

We examined the ability of Australian English- and North Holland Dutch-learning infants to detect changes to linguistic (vowel change) and to indexical information (change in speaker of the same accent, or of a different accent) to determine whether and to what extent infants attended to both types of information during speech perception. The aim was to identify the type of information in speech that infants are attentive to and that therefore is potentially available to inform their natural learning of vowel categories. Our results for the first trial block show that at 12 months, infants attend to indexical and linguistic differences in isolated vowel tokens: Infants’ looking times to the experimental trials that contained an Indexical change and those that contained a Vowel change were greater compared to their looking times to the No-change control trial. This finding shows that at this young age, infants, like adults, are sensitive to information that distinguishes speakers, and this corresponds to their emergent ability to recognize speech across different speakers and accents at around this age (e.g., [22,24]). Thus, our data are also consistent with the interpretation that infants are sensitive to indexical information at 12 months, which is a precondition for the use of these cues as contexts from which phonemic variation can be conditionalized.

Our results did not show a difference in looking time preferences at test between AusE- and NHD-learning infants. The study familiarized infants to the native NHD vowel /ɪ/ produced by a native NHD speaker, and tested listening preference to the native NHD vowel /ɛ/ produced by the same NHD speaker, /ɪ/ produced by a different NHD speaker, and/or /ɪ/ (which was acoustically similar to NHD /ɛ/) produced by a native EFD speaker. Thus, NHD-learning infants were familiarized to native vowels, and tested on native and variant vowels; AusE-learning infants were effectively familiarized and tested on variant (i.e., non-native) vowels in each case. The lack of an effect of language background here does not necessarily indicate that language background is unrelated to infants’ performance, but it does not support a native language advantage in the task. Moreover, if there were an overall novelty effect for the AusE-learning infants because they were tested on stimuli produced exclusively by native NHD speakers, one would expect an overall effect of group, which was not present in our findings.

Notably, infants’ performance cannot straightforwardly be attributed to overall magnitude differences in acoustic properties of our auditory stimuli. Two of our measures are typically regarded as reflecting voice quality (that could be associated with speaker identity), namely F0 and energy in high frequencies (corresponding to the pitch and breathiness of a speaker’s voice). The other measures, F1, F2 and F3, reflect acoustic properties of vowel categories, though listeners most likely associate these with speaker characteristics as well (e.g., low frequencies in general indicating a larger vocal tract than high frequencies). Apart from the measures we list in the Tables, there are other, more subtle cues in the speech signal on which listeners could rely when identifying a speaker or a vowel, and this is particularly likely when using natural speech, as was used here, rather than synthesized speech. For those reasons, we have not included any measures of perceptual/acoustic distance directly in our analyses. However, from the acoustic values that are listed in the Tables, it can be observed that the pattern of results cannot be attributed simply to the magnitude of the acoustic differences in F1-F3. Instead it appears to have been more likely due to differences in voice quality. This is because the two measures we have for voice quality (F0 and energy) yield comparable differences between Test and Control stimuli across both types of change (indexical vs. linguistic).

We cannot exclude the possibility therefore that infants did not (need to) access their linguistic levels of representation in this task. In such a case, it is plausible that the increase in looking time from the No-change trials to the Vowel-change trials and Indexical-change trials reflects a reaction to a new token purely based on the auditory differences between tokens. However, if infants were only reacting to new tokens, we might expect looking time to be the same across the Indexical-change and Vowel-change trials. While looking times between these trials were not different when compared directly, differences in looking times to the Indexical-change trials relative to the No-change trials during the first block were of a greater magnitude than difference in looking times between Vowel-change and No-change trials. Thus, even if there was no necessity to access linguistic levels of representation due the absence of lexical or phonemic context surrounding the isolated vowels, this does not eliminate the possibility that information other than token novelty affected the infants’ looking times. Ongoing research in our lab tests infants’ detection of linguistic and indexical vowel changes in an electroencephalography (EEG) task, which may provide a more sensitive measure of change detection than behavioural looking times, which are very variable in infants. If this method shows differences in detection of the various linguistic and indexical changes, this would allow us to rule out the interpretation that infants are reacting simply to auditory novelty.

Infants’ failure to normalize indexical variation is consistent with the proposal by Rost and McMurray [40,41] that uncovering invariant components to cue segmental qualities requires a critical level of exposure to variability. This proposal was based in part on their finding that 14-month-olds failed to discriminate the minimal pair BUK-PUK in a word learning task when exposed to stimuli produced by a single speaker (see also [42]), but improved if they were exposed to variability in the speech signal that did *not* cue the phonemic distinction, via presentation of the words produced by several speakers. Even younger infants, at 2 and 6 months of age, discriminate a vowel contrast when exposed to multiple speakers [13,21]. Rost and McMurray’s proposal may also explain why 7.5-month-olds can recognize words between different-gendered speakers when first provided with exposure to the speakers [23], but fail in a paradigm that does not provide them with such exposure [22]. Future research could address infants’ performance when presented with a wider range of indexical information. It may be that in such a case, infants more readily normalize speaker variation, at least within the native accent.

Notably, we did not find evidence that infants detected changes to speakers when they occurred within or between accents differently. This contrasts with findings in adults. Kriengwatana et al. [32,33] asked NHD-speaking adults to categorize the same stimuli as presented in the current study as tokens of the vowel /ɪ/ or /ɛ/. Adults correctly categorized tokens of /ɪ/ or /ɛ/ spoken by the same NHD speaker, and thus normalized speaker variation, but did not normalize accent variation, categorizing EFD tokens of /ɪ/ as /ɛ/. However, adults could normalize accent changes if given feedback in the form of semi-explicit instruction in a behavioral task, in which participants received feedback when their categorizations of vowels produced by the accented speaker did not align with the phonemic categorizations of that accent. This is in line with the model by Kleinschmidt and Jaeger [19] whereby variability in the speech signal can be compartmentalized into a range of indexical and linguistic sources based on exposure and constant adjustment of phonetic categories via distributional learning. While in this case feedback was explicit within the task, in a natural environment, feedback is thought to be provided through linguistic and nonlinguistic context. Thus, adults’ initial failure to normalize accented vowel tokens in Kriengwatana et al. [33] and infants’ detection of indexical changes here can be seen as a failure to compartmentalize accent-based variability that becomes possible with increased context and/or feedback. This provides some explanation for our pattern of findings here and a clear direction for future research.

An important distinction between the present study and the other studies discussed above is that there was no specificity of target in the present study. Rost and McMurray’s [40,41] findings specifically required infants to normalize speaker variability to detect segmental differences, and Kriengwatana et al. [32,33] required adults to normalize speaker and accent variability to detect vowel differences. It may be that as there was no task value in treating speaker, accent or vowel qualities differently, infants did not have any need to expend cognitive resources to normalize accent and speaker differences.

The present study is one of the first to directly compare 12-month-olds’ perception of speaker, accent and vowel changes in the same task. There is an exciting scope for further research in this field. First, we have shown that infants are sensitive to indexical (speaker and accent) changes and linguistic (vowel) changes in a task that presented no specific demands on vowel, speaker or accent sensitivity. Second, we did not find evidence that infants were more sensitive to indexical or linguistic information at this age. In a more directed task (e.g., novel word learning), infants may show differing performance, depending on their ability to use and contextualize these cues. Alternatively, in a non-lexical task, the separation of indexical and linguistic variability may not be clear and instead must be normalized within a lexical context (see [6]). We are currently extending this research by examining pre-attentive normalization of Dutch vowels between changes in sex, accent, speaker, and vowel by 12-month-old and adult speakers of Australian English in a typical EEG Mismatch Negativity paradigm.

Additionally, younger infants still acquiring vowels prior to 6 months of age might weigh linguistic and indexical cues differently compared to older infants. The direction of the difference is difficult to predict. On the one hand, younger infants might altogether ignore (or adjust the vowel space for) indexical cues that specify speaker identity (for instance, pitch, or other voice quality markers such as breathiness or creakiness), paying more attention to the acoustic properties such as resonating frequencies that mark vowel identity. On the other hand, speaker changes might be more salient to younger infants because they need to make a decision about whether to treat new speakers as unique sources for their target language, rather than generalizing across speakers. Thus, development of an early receptive vocabulary, which occurs around 6 to 7 months [43], may signal a decrease in attention to indexical cues, or a shift in the way that indexical information is attended to. The first few words in a child’s receptive lexicon may be at first indexically specified (e.g., reflective of “baby” produced only by the child’s mother), but acquisition of the word forms in a referential context may then trigger infants to generalize over some degree of between speaker variation when present in a lexical context. Indeed, in the absence of exposure, 7.5-month-olds are unable to recognize familiarized word forms across speakers of different genders, but can do so at 10.5 months [22], suggesting a developmental difference in the salience of indexical cues between those ages.

Finally, our study could be extended by looking for an effect of increasing or directed exposure to variability, as in Rost and McMurrary [40,41], or White and Aslin [44]. As proposed above, the distributional properties of variability along different dimensions may reveal changes in infants’ attention to these dimensions as normalization progresses. These and other studies would lay the groundwork for exploring the relationship between the varying salience of indexical and linguistic cues and distributional learning processes.

In conclusion, the present study reports that 12-month-old infants reliably show sensitivity to both indexical information and linguistic information when listening to familiar and unfamiliar vowel tokens, whether they are of native or non-native vowels. This generality of sensitivity (from relatively small differences between speakers of the same accent producing the same vowel, to relatively large differences between regional accents) suggests the availability of linguistic and indexical cues to children of this age, which is crucial to distributional learning of native categories conditionalized on properties of speakers, such as accent or gender. This suggests a mechanism that may be readily available in core early linguistic tasks such as mastering the native distribution of the vowel space, within and across accents. This mechanism of distributional cue-weighting, in the light of other experimental results, needs to be delineated with further investigation of the role of exposure. The experimental approach used here opens up wide avenues of research in terms of expansion to real-world tasks such as recognition and word learning, within- and cross-linguistically, to determine the role these sensitivities play in linguistic development and how infants cope with linguistic variation.

## Supporting information

S1 Conference Paper Published conference paper on preliminary data [45]. (PDF)

S1 Table Table of means and SDs for raw looking times to test trials in milliseconds. (PDF)

S2 Table Mean differences and SDs in looking time (ms) within-subject across test trial types for each block. (PDF)

S3 Table Within-subject correlations of mean looking times (Mean LT) of between-condition changes (Indexical-, Vowel-, and No-change) for each block. (PDF)
