# Supplementary material for: Indexical and linguistic processing by 12-month-olds: Discrimination of speaker, accent and vowel differences
Source: PLoS One. 2017 May 17;12(5):e0176762. doi: 10.1371/journal.pone.0176762 (PMC5435166; doi:10.1371/journal.pone.0176762)
Supplement: S1 Conference Paper — (PDF) [file pone.0176762.s001.pdf]

Indexical and linguistic processing in infancy: Discrimination of speaker, accent and vowel differences

Paola Escudero[1], Cory D. Bonn[2], Richard N. Aslin[2], Karen E. Mulak[1]
[1]University of Western Sydney, [2]University of Rochester
paola.escudero@uws.edu.au, cbonn@bcs.rochester.edu, aslin@bcs.rochester.edu, k.mulak@uws.edu.au

ABSTRACT

Infants preferentially discriminate native speech-sound categories prior to acquiring a large receptive vocabulary, implying a major role for distributional learning strategies in phoneme learning. However, it is unknown how infants extract the vowel phonemes of their language from distributional information in the presence of between-speaker variability in vowel realizations. Before we can ask this question, we must determine whether both indexical and linguistic cues are available to infants in speech processing.

We familiarized infants to tokens of a vowel produced by one speaker, and tested their listening preference to trials containing a vowel change produced by the same speaker (linguistic information), and the same vowel produced by a speakers of the same or a different accent (indexical information). Infants noticed linguistic and indexical differences, suggesting that both are salient in infant speech processing. Further research should explore how infants weight these cues in distributional learning of vowel categories.

Keywords: Infant speech processing; indexical vs. linguistic information; speaker vs. phoneme

1. INTRODUCTION

Though infants can understand a handful of common words by 6 months of age [5], it is generally accepted that this receptive vocabulary is insufficient for learning the phonemic distinctions relevant to their target language from minimal pairs alone. In other words, there are too few word forms in the lexicon for which infants know the referents (if any) and that also differ by only one phoneme to locate all of the native category boundaries or prototypes. In particular, studies using the conditioned head-turn procedure have shown that infants can discriminate the vowels of their native language at 6 months of age (i.e., show a perceptual magnet effect for category prototypes) [14], which is almost certainly too early for the developing lexicon to play a substantive computational role in organizing phonemic knowledge. Thus, the primary mechanism by which infants come to preferentially discriminate phonetic tokens sampled across native speech-sound category boundaries is thought to be unsupervised distributional learning over the raw statistics of the acoustic input [15]. We know that knowledge of word forms—independent of their meaning—could play a role in constraining distributional learning by providing contextual information that helps keep distributions separate, at least in 8-month-old infants [9].

However, all speech production, both across productions of individual words and across realizations of individual phonemes, is subject to variability in specific tokens within and across individual speakers. A current important question is how infants extract the phonemic categories of their native language from distributional information when the input contains between-speaker variability, such as is found between speakers of the same accent and speakers of different accents. While infants can discriminate speech sounds and recognize words produced by different speakers [13], [17], they have more difficulty doing so when the words are produced in an unfamiliar accent [1, 6, 10, 16].

In order to later examine how infants extract phonemic category information, in particular, vowel category information, from input containing between-speaker variation, we must first learn what cues (indexical [speaker, accent] and linguistic [phonemic distinctions]) are available to infants in distributional learning, and the relative salience of these cues. Given the assumption that infants can track the identity of speakers and the additional assumption that between-speaker variability is far greater than within-speaker variability in vowel production, it might be possible for infants to infer the location of distributions in acoustic space by correcting for speaker differences in acoustic realizations of vowel categories.

In the present study, we thus tested in detail whether infants are sensitive to both indexical and phonemic differences in speech sounds. The availability of these cues may help organize statistical information about vowels and speech categories more generally—an ability that appears to be important into adulthood [2]: In spite of tremendous variability in phoneme realizations across speakers of a specific accent and across speakers with different accents, adults seem to be

able to rapidly accommodate this variability with ease [1, 2, 10].

Specifically, we examined whether Australian English-learning and North Holland Dutch-learning infants notice differences in vowels produced by different speakers of North Holland Dutch accent, or a different accent of Dutch, East Flemish Dutch, to the same extent that they notice differences between vowel categories spoken by a single speaker of North Holland Dutch. Attention to these indexical and linguistic differences would indicate the possibility that these cues are available to infants for sociolinguistic as well as linguistic purposes. Additionally, by comparing performance across Australian English- and North Holland Dutch-learning infants, we investigated whether this ability applies cross-linguistically, regardless of variation in linguistic properties across languages (i.e., across languages that have different phonemes or different realizations of the same phonemes).

2. METHOD

2.1. Participants

Participants were 37 infants from households where North Holland Dutch was spoken (22 females, mean age: 12.2 months) and 36 infants from households where Australian English was spoken (15 females, mean age: 14.3 months). The North Holland Dutch-learning infants were tested at the University of Amsterdam and the Australian English-learning infants were tested at the University of Western Sydney.

2.2. Stimuli

Infants were presented with naturally produced Dutch vowels extracted from read sentences. The vowels were selected from a larger corpus of Dutch vowels reported in [3]. We chose the vowels /ɪ/ and /ɛ/ (as in "pit" and "pet") because they have large variation in their acoustic properties across both Dutch and English accents, thus providing a realistic context in which speaker and accent variability would be behaviorally relevant. Moreover, acoustic analyses, for example [7], suggest that Australian English does not have /ɛ/, but /e/, which is more acoustically similar to the Dutch /ɪ/ than /ɛ/.

Thus, we predicted based on previous work [8], [4], that this contrast should be as easy for Australian English-learning infants to detect as for North Holland Dutch-learning infants, despite the fact that it is a non-native contrast for the former. In addition, the fact that some of the Australian English-learning infants in the present study were older than the native North Holland Dutch-learning infants could compensate for any potential discrimination difficulty in perceiving non-native vowels.

The Dutch vowels presented to the infants in the present study were produced by two female speakers of the same Dutch accent (North Holland Dutch) and by a speaker of a different Dutch accent (East Flemish Dutch). Figure 1 shows the F1 and F2 values of these vowels. As can be observed, acoustic analysis of the stimuli confirmed that F1 and F2 values of /ɪ/ are most similar across speakers of the same accent than across speakers of different accents. Importantly, the values of East Flemish /ɪ/ were closer to those of North Holland /ɛ/ than to North Holland /ɪ/.

Figure 1: F1 and F2 values of the present study's stimuli. Black = North Holland Dutch; grey = East Flemish Dutch. Vowel tokens from the speaker used in familiarization (first North Holland Dutch speaker) are circled.

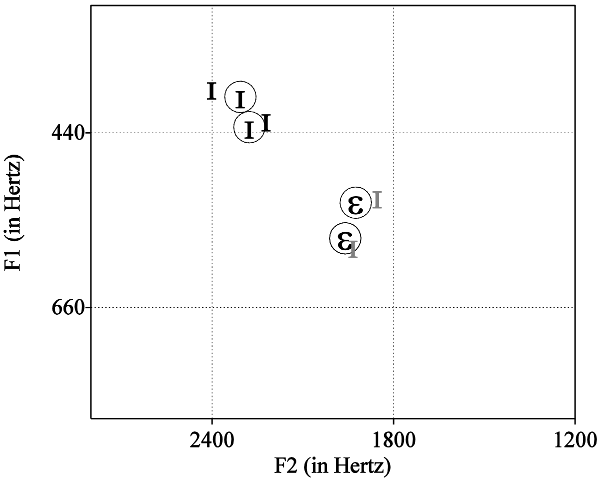

2.3. Procedure

In a serial preference procedure, we measured infants' looking times to trials composed of strings of vowel tokens. The different trial types presented to infants are summarized in Table 1.

Table 1: Vowel tokens for the familiarization and test trials. Tokens were produced by one of two female speakers of North Holland Dutch (NHD1, NHD2) or a female speaker of East Flemish Dutch (EFD).

Trial	Token alternation
Familiarization	/ɪ/-NHD1, /ɪ/-NHD1
Same	/ɪ/-NHD1, /ɪ/-NHD1
Vowel change	/ɪ/-NHD1, /ɛ/-NHD1
Speaker change	/ɪ/-NHD1, /ɪ/-NHD2
Accent change	/ɪ/-EFD, /ɪ/-EFD

Infants first heard eight familiarization trials containing two alternating tokens of /ɪ/ produced by one of the female North Holland Dutch speakers. Each familiarization trial contained eight repetitions of each /ɪ/ vowel token, for a total of 16 repetitions of /ɪ/, with a 750 ms inter-stimulus-interval, for a total trial duration of 13 sec. This procedure maximized the chances of inducing boredom in infants while maintaining a fixed-length exposure period. This means that while the procedure is technically a preferential looking procedure with a familiarization phase, it shares a strategy that is similar to studies that exploit habituation behavior. Thus, we anticipated using a change in looking duration from the average of the last two familiarization trials to each test trial as the dependent measure.

After familiarization, infants were presented with three test trials in random order. Test Trial Type 1 was the same as a familiarization trial (Same trial). Test Trial Type 2 contained alternating tokens of /ɪ/ and /ɛ/ spoken by the same North Holland female as in the familiarization phase (Vowel change trial).

For Test Trial Type 3, 35 infants heard alternating tokens of /ɪ/ produced by the familiarization speaker and by the other female speaker of North Holland Dutch (Speaker change trial), while the remaining 38 infants heard alternating tokens of /ɪ/ produced by the familiarization speaker and by the speaker of East Flemish Dutch (Accent change trial).

Stimuli were presented using E-Prime 2.0, and infants' looking times were collected using a Tobii X120 eye-tracker sampling at 120Hz.

3. RESULTS

We first calculated difference scores between looking times to test trials and the average of the last two familiarization trials. Figure 2 shows the difference scores for the three types of test trials for each language group.

Difference scores were entered as the dependent variable of a repeated measures ANOVA with Test Trial (same, vowel, speaker/accent change) as the within-subject variable and infants' native language (North Holland Dutch vs. Australian English) and infants' Test Trial Type 3 (Speaker vs. Accent change) as between-subjects factors.

This analysis revealed a main effect of test trial, $F(2, 138) = 6.65$, $p = .002$, $\eta_p^2 = .088$. Planned paired comparisons confirmed that infants had a larger difference in looking time to the Vowel change trial, $t(72) = 2.26$, $p = .027$, and to the Speaker/Accent change trial, $t(72) = 3.45$, $p = .001$ than the Same trial, but that looking time did not differ between Vowel and Speaker/Accent change trials, $t(72) = 1.30$, $p = .198$. There was no difference between language groups, as neither the main effect of native language nor any of the interactions involving native language were significant (all *ps* > .25).

Figure 2: Difference in looking time to test trials relative to the last two familiarization trials for Australian English (AusE)-learning and North Holland Dutch-learning (NHD) infants. Error bars represent one standard error.

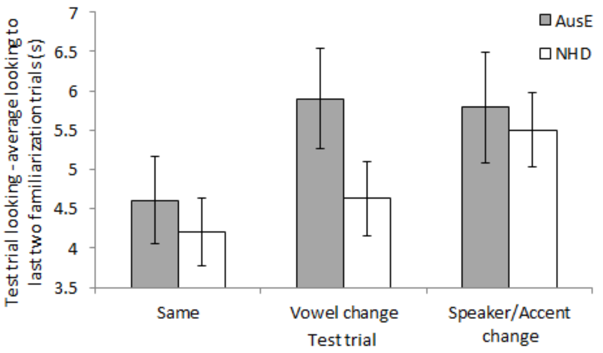

4. DISCUSSION

We compared Australian English- and North Holland Dutch-learning infants' detection of linguistic changes (vowel phoneme change) and changes to indexical information (change in the speaker and/or the speaker's accent) in vowel production to determine whether infants attended to both types of information during speech perception. Our results show that infants can notice linguistic and indexical differences in vowel production to similar extents, indicated by their greater difference in looking time to Vowel change test trials and Speaker or Accent change test trials from the last two familiarization trials, relative to control trials. This suggests that at this young age, infants are able to distinguish the same type of information as adults [1, 2, 10].

We are currently testing more 12-month-old infants in the Australian population to better match the age range of the Dutch infants, though the age differences here do not seem to affect the results. This is likely due to the fact that all ages tested are beyond the age range at which perceptual narrowing is thought to occur for vowels.

Our findings demonstrate that by 12 months, infants have access to both linguistic and indexical information in situations of between-speaker variability, suggesting that both cues would be available to them for distributional learning of vowel categories. Further research should explore how weight given to each type of acoustic cue varies by task and across varying stages of phoneme

acquisition from 3-12 months of age. One possibility is that they might ignore some of these differences in more linguistic tasks involving vowel categorization (e.g., conditioned head-turn procedure).

In equivalent, age-appropriate tasks, adults tend to ignore indexical differences [12]. This asymmetry in how adults weight indexical and phonemic cues appears to emerge during development after 6 months of age. Introducing greater variance in the tokens in familiarization along either an indexical dimension or a linguistic dimension may have different kinds of effects on preferential looking behavior during test trials.

Another possibility is that cue weights may vary as a function of learning stage: Younger infants still acquiring vowels prior to 6 months of age might weight linguistic and indexical cues differently compared to older infants. The direction of the difference is difficult to predict. On the one hand, they might ignore (or adjust the vowel space for) indexical cues altogether, paying more attention to lower frequency information. On the other hand, speaker changes might be more salient to younger infants because they need to make a decision about whether to treat new speakers as informative sources for their target language or not. Indeed, in the absence of exposure, 7.5-month-olds are unable to recognize familiarized word forms across speakers of different genders, but can do so at 10.5 months [11], suggesting a developmental difference in the salience of indexical cues between those ages.

Further research on infants' processing of linguistic and indexical speech cues would lay the groundwork for exploring the relationship between the varying salience of those cues and distributional learning processes. It could be that infants can use knowledge about noise levels and statistical structure in individual phoneme productions to constrain their expectations about how groups of phonemes are realized in novel speakers. Alternatively, the ability to generalize learned distributions across speakers may come later in childhood: In this case, infants should only learn phonemes for which distributions of tokens can be reliably separated in acoustic space.

7. REFERENCES

[1] P. Adank, B. G. Evans, J. Stuart-Smith, and S. K. Scott, "Comprehension of familiar and unfamiliar native accents under adverse listening conditions.," *J. Exp. Psychol. Hum. Percept. Perform.*, vol. 35, no. 2, pp. 520–529, 2009.

[2] P. Adank and J. M. McQueen, "The effect of an unfamiliar regional accent on spoken word comprehension," in *Procedings of the XVIth International Congress of Phonetic Sciences*, Saarbrucken, Germany, 2007, pp. 1925–1928.

[3] P. Adank, R. van Hout, and R. Smits, "An acoustic description of the vowels of Northern and Southern Standard Dutch," *J. Acoust. Soc. Am.*, vol. 116, no. 3, pp. 1729–1738, Sep. 2004.

[4] S. Alispahic, P. Escudero, and K. E. Mulak, "Is more always better? The perception of Dutch vowels by English versus Spanish listeners.," in *Proceedings of the 15th Australasian International Conference on Speech Science and Technology*, Singapore, 2014, pp. 1293–1296.

[5] E. Bergelson and D. Swingley, "At 6–9 months, human infants know the meanings of many common nouns," *Proc. Natl. Acad. Sci.*, vol. 109, no. 9, pp. 3253–3258, Feb. 2012.

[6] C. T. Best, M. D. Tyler, T. N. Gooding, C. B. Orlando, and C. A. Quann, "Development of phonological constancy: Toddlers' perception of native- and Jamaican-accented words," *Psychol. Sci.*, vol. 20, no. 5, pp. 539–542, 2009.

[7] F. Cox, "Australian English pronunciation into the 21st century," *Prospect*, vol. 21, no. 1, pp. 3–21, 2006.

[8] P. Escudero, C. T. Best, C. Kitamura, and K. E. Mulak, "Magnitude of phonetic distinction predicts success at early word learning in native and non-native accents," *Lang. Sci.*, vol. 5, p. 1059, 2014.

[9] N. H. Feldman, E. B. Myers, K. S. White, T. L. Griffiths, and J. L. Morgan, "Word-level information influences phonetic learning in adults and infants," *Cognition*, vol. 127, no. 3, pp. 427–438, Jun. 2013.

[10] C. Floccia, J. Goslin, F. Girard, and G. Konopczynski, "Does a regional accent perturb speech processing?," *J. Exp. Psychol. Hum. Percept. Perform.*, vol. 32, no. 5, pp. 1276–1293, Oct. 2006.

[11] D. M. Houston and P. W. Jusczyk, "The role of talker-specific information in word segmentation by infants," *J. Exp. Psychol. Hum. Percept. Perform.*, vol. 26, no. 5, pp. 1570–1582, 2000.

[12] B. Kriengwatana, P. Escudero, and J. Terry, "Listeners cope with speaker and accent variation differently: Evidence from the Go/No-go task. *Proceedings of SST 2014, Christchurch, New Zealand.*," in *Proceedings of the 15th Australasian International Conference on Speech Science and Technology*, Singapore, 2014, pp. 76-79.

[13] P. K. Kuhl, “Perception of auditory equivalence classes for speech in early infancy,” *Infant Behav. Dev.*, vol. 6, no. 2–3, pp. 263–285, 1983.

[14] P. K. Kuhl, K. Williams, F. Lacerda, K. Stevens, and B. Lindblom, “Linguistic experience alters phonetic perception in infants by 6 months of age,” *Science*, vol. 255, no. 5044, pp. 606–608, Jan. 1992.

[15] J. Maye, J. F. Werker, and L. Gerken, “Infant sensitivity to distributional information can affect phonetic discrimination,” *Cognition*, vol. 82, no. 3, pp. B101–B111, 2002.

[16] K. E. Mulak, C. T. Best, M. D. Tyler, C. Kitamura, and J. R. Irwin, “Development of phonological constancy: 19-month-olds, but not 15-month-olds, identify words spoken in a non-native regional accent,” *Child Dev.*, vol. 84, no. 6, pp. 2064–2078, 2013.

[17] M. van Heugten and E. K. Johnson, “Infants exposed to fluent natural speech succeed at cross-gender word recognition,” *J. Speech Lang. Hear. Res.*, vol. 55, no. 2, pp. 554–560, Apr. 2012.
