# Supplementary material for: Indexical and linguistic processing by 12-month-olds: Discrimination of speaker, accent and vowel differences
Source: PLoS One. 2017 May 17;12(5):e0176762. doi: 10.1371/journal.pone.0176762 (PMC5435166; doi:10.1371/journal.pone.0176762)
Supplement: S1 Table — (PDF) [file pone.0176762.s002.pdf]

Participants' native language	Indexical change condition	Block	Test trial type	Mean	SD
Australian English	Speaker	1	Indexical change	5629.92	3351.96
Australian English	Speaker	2	Indexical change	5321.66	3001.48
Australian English	Speaker	1	Vowel change	5714.55	3022.90
Australian English	Speaker	2	Vowel change	5007.24	3475.00
Australian English	Speaker	1	No change	4989.29	3099.41
Australian English	Speaker	2	No change	4229.14	3358.93
Australian English	Accent	1	Indexical change	6825.42	3791.45
Australian English	Accent	2	Indexical change	5411.81	3254.68
Australian English	Accent	1	Vowel change	6570.74	3650.87
Australian English	Accent	2	Vowel change	5765.40	3544.85
Australian English	Accent	1	No change	6003.14	3431.38
Australian English	Accent	2	No change	5160.25	3371.79
North Holland Dutch	Speaker	1	Indexical change	6456.83	2454.54
North Holland Dutch	Speaker	2	Indexical change	3444.73	1857.02
North Holland Dutch	Speaker	1	Vowel change	4982.13	2300.26
North Holland Dutch	Speaker	2	Vowel change	3998.00	1764.89
North Holland Dutch	Speaker	1	No change	5042.96	2656.41
North Holland Dutch	Speaker	2	No change	4053.36	1773.52
North Holland Dutch	Accent	1	Indexical change	5178.94	3016.38
North Holland Dutch	Accent	2	Indexical change	3658.03	2626.31
North Holland Dutch	Accent	1	Vowel change	4744.65	3226.81
North Holland Dutch	Accent	2	Vowel change	3634.43	3142.72
North Holland Dutch	Accent	1	No change	4010.32	2295.93
North Holland Dutch	Accent	2	No change	4053.72	3192.46
