# Supplementary material for: Indexical and linguistic processing by 12-month-olds: Discrimination of speaker, accent and vowel differences
Source: PLoS One. 2017 May 17;12(5):e0176762. doi: 10.1371/journal.pone.0176762 (PMC5435166; doi:10.1371/journal.pone.0176762)
Supplement: S2 Table — (PDF) [file pone.0176762.s003.pdf]

Participants' native language	Indexical change condition	Block	Contrast	Mean	SD
Australian English	Speaker	1	Indexical change vs. No change	640.63	2264.33
Australian English	Speaker	1	Indexical change vs. Vowel change	-84.63	2341.78
Australian English	Speaker	1	Vowel change vs. No change	725.26	2191.53
Australian English	Speaker	2	Indexical change vs. No change	884.96	2929.22
Australian English	Speaker	2	Indexical change vs. Vowel change	27.06	3716.85
Australian English	Speaker	2	Vowel change vs. No change	894.31	2422.28
Australian English	Accent	1	Indexical change vs. No change	822.28	2555.94
Australian English	Accent	1	Indexical change vs. Vowel change	254.67	2840.73
Australian English	Accent	1	Vowel change vs. No change	567.61	3036.38
Australian English	Accent	2	Indexical change vs. No change	251.56	2825.58
Australian English	Accent	2	Indexical change vs. Vowel change	-362.67	3064.18
Australian English	Accent	2	Vowel change vs. No change	614.24	3337.16
North Holland Dutch	Speaker	1	Indexical change vs. No change	1413.86	3671.71
North Holland Dutch	Speaker	1	Indexical change vs. Vowel change	1474.69	2250.54
North Holland Dutch	Speaker	1	Vowel change vs. No change	-60.83	2703.47
North Holland Dutch	Speaker	2	Indexical change vs. No change	-608.63	2374.08
North Holland Dutch	Speaker	2	Indexical change vs. Vowel change	-819.80	1780.29
North Holland Dutch	Speaker	2	Vowel change vs. No change	211.17	2172.30
North Holland Dutch	Accent	1	Indexical change vs. No change	1168.62	2753.05
North Holland Dutch	Accent	1	Indexical change vs. Vowel change	434.29	2569.49
North Holland Dutch	Accent	1	Vowel change vs. No change	734.32	2663.41
North Holland Dutch	Accent	2	Indexical change vs. No change	-395.70	2816.07
North Holland Dutch	Accent	2	Indexical change vs. Vowel change	23.60	2681.22
North Holland Dutch	Accent	2	Vowel change vs. No change	-419.29	3027.06
