# Supplementary material for: Indexical and linguistic processing by 12-month-olds: Discrimination of speaker, accent and vowel differences
Source: PLoS One. 2017 May 17;12(5):e0176762. doi: 10.1371/journal.pone.0176762 (PMC5435166; doi:10.1371/journal.pone.0176762)
Supplement: S3 Table — (PDF) [file pone.0176762.s004.pdf]

BLOCK 1	Indexical change vs. No change	Vowel change vs. No change	Indexical change vs. Vowel change
Indexical change vs. No change	1.00	0.56	0.51
Vowel change vs. No change		1.00	-0.42
Indexical change vs. Vowel change			1.00
**BLOCK 2**			
Indexical change vs. No change	1.00	0.50	0.48
Vowel change vs. No change		1.00	-0.52
Indexical change vs. Vowel change			1.00
